# Point-of-Care Ultrasonography in the Critical Care Unit: An Update

**DOI:** 10.1007/s11886-024-02187-3

**Published:** 2025-02-15

**Authors:** Keith Guevarra, Yonatan Greenstein

**Affiliations:** Department of Medicine, Division of Pulmonary and Critical Care Medicine, Rutgers – New Jersey Medical School, University Hospital Building, 150 Bergen Street, Newark, NJ 07103 USA

**Keywords:** Critical care ultrasonography, Point-of-care ultrasonography, Echocardiography, POCUS, Artificial intelligence, Update

## Abstract

**Purpose of Review:**

This article outlines updates in point of care ultrasonography.

**Recent Findings:**

Improving diagnostic accuracy and image quality is continuing to evolve in Point-of-care ultrasonography (POCUS). This include incorporating Artificial Intelligence (AI) and use of other modalities such as Doppler in lung ultrasonography. Transesophageal echocardiography is an emerging option when imaging is difficult to obtain via transthoracic echocardiography. POCUS is becoming instrumental when used during cardiac arrest. Ultrasound (VExUS) Grading System is a promising measurement to assess a patient’s volume status. Given the multiple advantages of POCUS usage, competency in POCUS became a requirement of Critical Care fellowship training.

**Summary:**

POCUS is an important diagnostic modality and guide to medical management. New technological advances such as AI, can serve as a guide to enhance image quality and help accurately obtain quantitative assessments. POCUS has a major role during cardiac arrest and advanced cardiac life support. The clinical utility of POCUS was further substantiated during the COVID-19 pandemic. The Accreditation Council for Graduate Medical Education (ACGME) requires critical care programs to include competency in POCUS as part of their training.

**Supplementary Information:**

The online version contains supplementary material available at 10.1007/s11886-024-02187-3.

## Introduction

Point-of-care ultrasonography (POCUS) is an important diagnostic tool in the critical care unit. In 2020, we published a broad overview of POCUS in this Journal [1] and in this paper we review the latest updates to keep frontline clinicians abreast of cutting-edge applicable research and policies. This review will focus on emerging techniques and technologies and the new mandated ACGME requirements for critical care fellowship training programs.

## Technological Advances

During the past 30 years of POCUS use in critical care medicine, image acquisition and interpretation has relied on sufficient training and skill of the operator. As a result, the skillset of critical care POCUS users can vary significantly. Basic POCUS users rely on a simplified qualitative assessment that includes inherent assumptions that are not always correct [2]. For example, a hypotensive patient with a severe reduction in left ventricular systolic function may be thought to have cardiogenic shock. An advanced POCUS user will include a quantitative assessment of cardiac output to more accurately determine the type of shock the patient is in. Accuracy of measurements are highly dependent on the scanner’s expertise in image acquisition and Doppler physics.

Rapid advances in machine learning and artificial intelligence (AI) are beginning to lower the entry bar to basic POCUS and advanced quantitative measurements. Several commercially available POCUS devices give real-time image quality feedback. Thus, a novice scanner has the potential to acquire high quality images with the aid of AI. Some POCUS devices now include the ability to automatically perform advanced quantitative assessments. In a study by Gohar et al., AI in a handheld ultrasound transducer was able to calculate left ventricular (LV) ejection fraction, LV outflow tract velocity time integral, and inferior vena cava size and collapsibility. The machine was also able to provide user feedback regarding the quality of the acquired image. Agreement was good between an expert sonographer’s manual measurement and the AI measurement, as long as the views were of high or medium quality [3]. In a multicenter study of limited echocardiograms in COVID-19 patients, AI technology identified 50% of patients with a left ventricular ejection fraction of < 50%. The sensitivity of AI in clinical LVEF < 50% detection was 85.7% and specificity of 78.6%. The authors concluded that AI technology can provide accurate measurements in less time and can help decrease the need for bedside expertise [4]. More outcome studies are warranted to determine the impact of cardiac echocardiography aspect of POCUS using AI technology.

## Lung Ultrasonography

Lung ultrasonography is routinely used to determine the etiology of respiratory failure and to guide management. Readers unfamiliar with the use of this modality are referred to our prior paper [1].

It is difficult for a clinician to accurately distinguish the etiology of B-lines based on the appearance of lung ultrasonography. A smooth pleural line suggests hydrostatic pulmonary edema over infectious or inflammatory causes, while a thickened or irregular pleural line with reduced lung sliding and small peripheral consolidations suggest an infectious or inflammatory etiology [5, 6] (Video 1 and 2). Researchers have begun to leverage AI for the analysis of lung ultrasound and studies are extremely promising. Arntfield and colleagues used AI to identify subvisible ultrasound features, allowing the software to distinguish COVID-19, non-COVID-19, and hydrostatic pulmonary edema as the cause of B-lines (overall area under the curve 0.789 for humans vs. 0.978 for the AI model) [7].

Distinguishing pneumonia from atelectasis with lung ultrasonography has been challenging. Clinicians must integrate the clinical context into the imaging findings. Dynamic air bronchograms were found to be highly specific for pneumonia [8]. Their sensitivity, however, is lacking. Several studies have explored the potential for color Doppler to aid in distinguishing pneumonia from atelectasis [9, 10]. Recently, Haaksma et al. studied the utility of diagnosing pneumonia with lung ultrasound by identifying the presence of dynamic air bronchograms or the presence of pulsatile color Doppler flow of the consolidated lung when static air bronchograms were present. They found that dynamic air bronchograms have a high positive predictive value (96%) and color Doppler had high negative predictive value (90%) [11]. This approach is promising and warrants further research.

## Cardiac Arrest

**Fig. 1:**
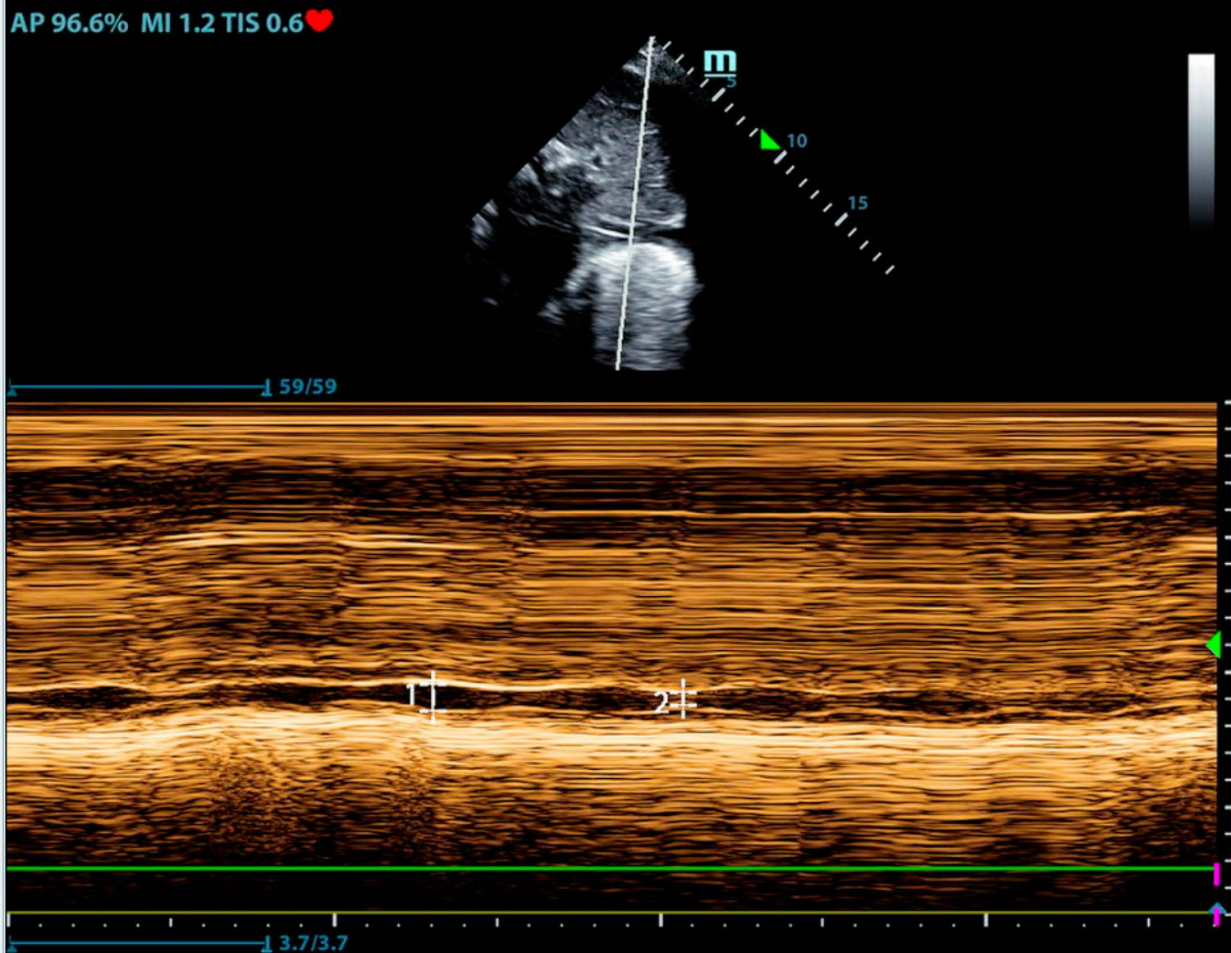
Collapsed IVC suggestive of hypovolemia

We previously outlined our use of POCUS for the evaluation of patients with undifferentiated shock [1]. The utility of POCUS extends beyond shock to the management of patients during a cardiac arrest. Standard advanced cardiac life support (ACLS) is algorithmic, and clinicians are tasked with identifying reversible causes of the cardiac arrest, the so called “H’s and T’s,” during the resuscitation. Hypovolemia (Video 3, Video 4 and Fig. 1), tension pneumothorax (Video 5), thrombosis (Video 6 and 7), and cardiac tamponade, all have the potential to be identified with POCUS performed during the resuscitation.

Multiple publications have shown the utility of POCUS in cardiac arrest. In addition to identifying reversible causes of the arrest, POCUS may provide prognostic information. Gaspari et al. showed that cardiac activity on initial ultrasound during ACLS compared to no cardiac activity was associated with higher return of spontaneous circulation (ROSC) and survival to hospital admission (28.9% vs. 7.2%) and higher survival to hospital discharge (3.8% vs. 0.6%) [12]. Patients that had an absence of cardiac activity on POCUS and asystole on the monitor had a 5.9% survival rate to hospital admission. A single center observational study found that cardiac arrest patients, with cardiac activity seen with POCUS, received a longer duration of ACLS resuscitation than patients with absence of cardiac activity on POCUS (27.33 min vs. 11.51 min) and a longer duration than those who were not assessed for cardiac activity by POCUS (14.36 min,* P* value < 0.001). Administration of epinephrine was more frequent in the POCUS group with cardiac activity compared to patients without cardiac activity and those who never received POCUS (100% vs. 82.39% vs. 81.39% *P* value < 0.001, respectively). Furthermore, a higher proportion of patients were endotracheally intubated in the group with cardiac activity detected with POCUS compared to absent cardiac activity detected with POCUS and patients that did not have POCUS performed (95.63% vs. 46.54% vs. 65.11% *P* value < 0.001, respectively). This ultimately led to a greater chance in patients with POCUS-detected cardiac activity of ROSC (76.19% vs. 19.5% vs. 39.5% *P* value < 0.001, respectively) and survival to hospital admission (33.3% vs. 6.9% vs. 27.9% *P* value < 0.001, respectively). There was no statistical difference in survival to hospital discharge between the 3 groups [13]. A clinical trial whereby the treating clinical team is blinded to the POCUS findings would need to be performed to remove the inherent bias in this study where clinician belief of the POCUS findings could drive the decision to continue or terminate resuscitation.

Clinicians performing POCUS during a cardiac arrest must make sure that they do not interrupt high quality ACLS. In a study using a manikin POCUS simulator, pulmonary and critical care medicine fellows and emergency medicine residents were able to obtain good to excellent quality echocardiography images 83% of the time without interrupting ACLS resuscitation [14]. The participants made the correct echocardiographic diagnosis in 68% of cases and took an average 1.5 pulse checks to do so. This simulator study shows that POCUS integration during ACLS is possible; however, when utilized for real world clinical care, multiple factors can hinder image acquisition. The patient’s body habitus, presence of defibrillator pads or dressings on the chest, and ongoing chest compressions may negatively impact the diagnostic yield and reliability of this modality [15]. Transesophageal echocardiography (TEE) overcomes these obstacles and enables image acquisition and interpretation while high quality chest compressions are being delivered. In a small single center study of TEE use in the emergency department during ACLS or immediately post ROSC, a resuscitative four view exam was achievable in all patients [16]. The authors found that the area of maximal compression was optimal in only 47% of examined subjects and in 53% of subjects the area of maximal compression was over the left ventricular outflow tract or aortic root, likely negatively impacting the ACLS effort. Current cardiopulmonary resuscitation (CPR) guidelines recommend limiting pulse checks to no more than 10 s. Manual palpation of the carotid artery pulse can be unreliable and slow. In a study by Kang et al., POCUS to identify the carotid pulse was quicker than manual palpation (1.62 s vs. 3.50 s *P* < 0.001) [17]. We feel strongly that clinicians should be integrating POCUS into their routine ACLS care.

## Advanced Critical Care Echocardiography

### Transesophageal Echocardiography

In our previous paper we described the importance of transesophageal echocardiography (TEE) for the intensivist when transthoracic echocardiography is not feasible. This application is supported by the American College of Cardiology and the American Heart Association [18] and it is well established that trained intensivists and trained emergency physicians can safely and accurately perform critical care TEE [19–26]. This year, the National Board of Echocardiography has added an additional certification in TEE for intensivists that are getting board certified or are already board certified in Critical Care Echocardiography via the CCEeXAM (Special Competence in Critical Care Ultrasonography). This is a welcome addition that will help intensivists incorporate TEE into their toolbox.

## Assessment of Volume Status

Since the publication of the Beaubien-Souligny article [27], many clinicians have begun incorporating Venous Excess Ultrasound (VExUS) into their clinical care. This measurement includes measurement of the IVC diameter in addition to pulsed wave Doppler measurement of the portal, hepatic and intrarenal veins [28]. A PubMed search on 7/17/24 identified 84 published articles on VExUS. None of these studies are randomized prospective trials that evaluate the score and its integration in clinical care. A prospective observational study demonstrated that the baseline VExUS score was poorly predictive of response to diuretic-induced fluid depletion [29]. Another prospective observational study did not demonstrate an association of the VExUS score with fluid balance [30]. We look forward to reviewing prospective studies using VExUS to guide clinical care in a generalizable population and applaud the authors on this innovative concept; however, we caution clinicians on incorporating this score into clinical care until more robust research to support it is available.

## Utility of POCUS During the COVID-19 Pandemic

The COVID-19 pandemic which resulted in more than one million deaths in the United States was an accelerant for POCUS adoption at many institutions that had been slow to formally adopt POCUS into practice. As resources and staff were constrained, the benefits of POCUS were incontrovertible. Prior to this, a gradual acceptance of this modality has been unfolding. In 2003 the American Society of Echocardiography (ASE) considered POCUS a tool that extended the accuracy of bedside physical examination [31]. They recommended that clinicians performing it for patient care should have at least level 2 echocardiography training required (150 examinations performed, 300 interpreted) and they held the user accountable for appropriate training, application, documentation, and interpretation of the data. In 2013, the ASE began using the term “focused cardiac ultrasound” and continued to distinguish it as a bedside adjunct to the physical exam compared to comprehensive echocardiography. They recommended not using any term that includes “echocardiography” to describe this application and they recommended that all patients with new abnormal findings be referred for comprehensive echocardiography [32]. The following year this requirement was changed when the ASE released an international statement which recommended that findings that were beyond the scope of the exam be referred for comprehensive echocardiography [33]. After the National Board of Echocardiography (NBE) offered board certification in Critical Care Echocardiography, the ASE released guidelines for echocardiography labs participating in POCUS training [34]. In this statement they use terminology different from prior position papers: ultrasound assisted physical examination, cardiac POCUS, critical care echocardiography (CCE), and standard transthoracic echocardiography. They indicate that CCE may employ many modalities common to standard transthoracic echocardiography (TTE), but they distinguish limited TTE from CCE by qualifying that limited TTE can be converted to a comprehensive study as needed. This evolution clearly recognizes the importance of POCUS while attempting to delineate the difference between cardiology performed echocardiography and echocardiography performed within POCUS or CCE.

In 2023, the ASE released a statement to guide clinicians during the COVID-19 pandemic and future pandemics [35]. They recommended that echocardiography labs avoid denying an appropriate echocardiogram solely based on the patient’s COVID-19 status. However, they recommended that limited echocardiography be performed to reduce exposure time to sonographers. These two statements might be considered in opposition of each other, especially when viewed through the lens of prior POCUS statements. Importantly, they stress that existing collaborative programs between cardiology programs and non-cardiologist POCUS and CCE clinicians are essential for better preparedness for the next pandemic. We see a future where all disciplines that perform overlapping ultrasound examinations come together at regular intervals for quality assurance, quality improvement, and education. Rather than the old model of sitting in silos, a collaborative approach will provide the most benefit to our patients.

## Training, Competence, Credentialing

In 2019, the NBE offered the first Examination of Special Competence in Critical Care Echocardiography. This board examination is one component in a pathway to becoming board certified in Critical Care Echocardiography. In addition to passing this examination, the applicant must complete 150 full critical care echocardiograms that are reviewed by a qualified physician. This year marks the first year that intensivists can also add TEE to this certification. As of the publication of this manuscript, approximately 1,700 physicians have taken the examination with an overall pass rate of 80%. There are approximately 1,360 testamurs of the examination, which is 7% of the intensivist workforce in the US. To date, 300 have achieved full board certification. Many current applicants have completed the certification requirements during fellowship training and the number of applicants and board-certified intensivists is expected to rise significantly.

On July 1st, 2024, the ACGME instituted sweeping new requirements that effect critical care training programs (Table 1) [36]. Whereas programs previously were only required to impart general awareness of point-of-care ultrasonography, they are now required to ensure that fellows can “demonstrate competence in the ability to” perform “those skills essential to critical care ultrasound, including image acquisition, image interpretation at the point of care, and use of ultrasound to place intravascular and intracavitary tubes and catheters.” Cardiology critical care fellowship programs will need to provide their trainees with non-cardiac POCUS training and program directors should refer to existing critical care ultrasonography training statements for guidance [37, 38].

**Table 1:** Ultrasound requirements based on Accreditation Council for Graduate Medical Education (ACGME) program requirements for graduate medical education in critical care medicine [36]

• IV.B.1.b).(2) Fellows must be able to perform all medical, diagnostic, and surgical procedures considered essential for the area of practice. **(Core)**
o IV.B.1.b).(2).(a) Fellows must demonstrate competence in the ability to: ▪ IV.B.1.b).(2).(a).(i) perform diagnostic and therapeutic procedures relevant to their specific career paths; **(Core)**
o IV.B.1.b).(2).(a) Fellows must demonstrate competence in procedural and technical skills, including: ▪ IV.B.1.b).(2).(c).(xi) technical and procedural skills of critical care ultrasound, including image acquisition, image interpretation at the point of care, and use of ultrasound to place intravascular and intracavitary tubes and catheters; (**Core**)
• IV.B.1.c) Medical Knowledge Fellows must demonstrate knowledge of established and evolving biomedical, clinical, epidemiological and social behavioral sciences, including scientific inquiry, as well as the application of this knowledge to patient care. (**Core**) ▪ IV.B.1.c).(1).(c) imaging techniques commonly employed in the evaluation of patients with critical illness, including the technical and procedural use of ultrasound and interpretation of ultrasound images at the point of care for medical decision making; (**Core**)

We applaud these new requirements but recognize that many critical care training programs will not be able to fulfill their obligation without significant investment. In 2019 Chulani et al. published results of a survey of academic critical care training program directors (PD). The findings shed light on the work to be done. The overall response rate was low at 16.5%. 82% of PD had no summative evaluation of fellow skills, 74% reported that faculty did not have protected time for teaching POCUS, and only 60% considered POCUS teaching as an important consideration for new hires. In terms of clinical integration, only 17% stored the majority of studies in the electronic record and 14% generated formal reports [39]. A survey of POCUS use in Department of Veteran Affairs ICUs with excellent response rates reported the following barriers to POCUS use: lack of trained providers (48%), lack of funding for training (45%), lack of training opportunities (37%), and lack of image archiving (35%) [40]. Critical care training programs will thus need to focus their resources on developing a comprehensive POCUS program that can efficiently train its fellows and assure high quality use for clinical care.

## Conclusions

Point of care ultrasonography is an important diagnostic modality. The noncardiac aspect of POCUS adds additional information that can help narrow the differential diagnosis and guide management. When TTE imaging quality is poor or not feasible to perform, TEE has major applications to guide diagnosis and management of critically ill patients. Artificial intelligence is beginning to show real world promise to aid clinicians in image acquisition and interpretation of ultrasound findings. Healthcare systems must work to build a collaborative multidisciplinary program for POCUS with a special focus on caring for patients during the next pandemic.

## Key References

- Gohar E, Herling A, Mazuz M, Tsaban G, Gat T, Kobal S and Fuchs L. Artificial Intelligence (AI) versus POCUS Expert: A Validation Study of Three Automatic AI-Based, Real-Time, Hemodynamic Echocardiographic Assessment Tools. J. Clin. Med. 2023, 12, 1352.
  - Findings in this study showed the impact of AI in obtaining accurate measurements
- Muñoz F, Born P, Bruna M, Ulloa R, González C, Philp V, Mondaca R, Blanco JP, Valenzuela ED, Retamal J, Miralles F, Wendel-Garcia PD, Ospina-Tascón GA, Castro R, Rola P, Bakker J, Hernández G, Kattan E. Coexistence of a fluid responsive state and venous congestion signals in critically ill patients: a multicenter observational proof-of-concept study. Crit Care. 2024 Feb 19;28(1):52. 10.1186/s13054-024-04834-1. PMID: 38374167; PMCID: PMC10877871.
  - Findings in this article demonstrated that presence of venous congestion was not associated with fluid balance
- Schott C, Wetherbee E, Khosla R, Nathanson R, Williams J, Mader M, Haro E, Kellogg D, Rodriguez A, Proud K, Boyd J, Bales B, Sauthoff H, Basrai Z, Resop D, Lucas B, Restrepo M, Soni N. Current Use, Training, and Barriers to Point-of-Care Ultrasound Use in ICUs in the Department of Veterans Affairs. CHEST Volume 1, issue 2, 100012, September 2023
  - Findings from this study enumerated barriers to POCUS use

## Supplementary Information

Below is the link to the electronic supplementary material.

Supplementary file1 Irregular pleura with B lines visualized with phased array transducer (3GP 488 KB)

Supplementary file2 Irregular pleura seen with straight linear array transducer (3GP 279 KB)

Supplementary file3 Parasternal long axis cardiac view showing end systolic effacement (MOV 20015 KB)

Supplementary file4 End systolic effacement seen in parasternal short axis cardiac view (MP4 1782 KB)

Supplementary file5 Lung point diagnostic of pneumothorax (MOV 27902 KB)

Supplementary file6 Apical 4 chamber view showing RV dilation (MOV 24849 KB)

Supplementary file7 Non-compressible common femoral vein with a thrombus (MP4 1838 KB)

## Data Availability

No datasets were generated or analysed during the current study.
